# Simple Auricular Hematoma (“Cauliflower Ear”) Task Trainer

**DOI:** 10.5070/M5.52387

**Published:** 2026-07-31

**Authors:** Laura Bailey, William Shaffer, Byron Thatcher, Aubrey Bethel-Schmitz

**Affiliations:** * Dignity Health East Valley, Department of Emergency Medicine, Chandler, AZ; ^ Creighton University Sc, Department of Emergency Medicine, Phoenix, AZ; † Creighton University School of Medicine – Phoenix

## Abstract

**Audience:**

This auricular hematoma task trainer is designed to instruct emergency medicine (EM) residents, fellows, and medical students. Otolaryngology trainees may also benefit from this simulator.

**Introduction:**

Auricular hematomas are relatively infrequent but require timely intervention to prevent complications such as infection, cartilage necrosis, or permanent ear deformity (“cauliflower ear”).^1^ Although these injuries make up only a small part of all otologic complaints in the emergency department, they are one of the most frequent ear injuries in contact sports, with wrestlers, boxers, and rugby players being the most at-risk.^2^ Not only can they occur in sports, one urban level I trauma review found 42% of auricular hematoma presentations were due to interpersonal violence or assault,^3^ making timely drainage a core skill now listed in the 2022 Model of the Clinical Practice of Emergency Medicine.^4^ EM residents often have limited opportunities to gain hands-on experience with this procedure in a controlled learning environment, with a low reported baseline confidence level highlighting a persistent training gap.^5^ Traditional models are expensive and single-use, with each one costing up to $60–100.^6^ This innovation was developed to address a training gap by providing a simple, affordable, and reproducible task trainer that enables learners to practice landmark identification, drainage, and post-procedure care of auricular hematomas with confidence.

**Educational Objectives:**

By the end of this session, learners will be able to: 1) identify the landmarks used to drain auricular hematomas, 2) demonstrate the ability to numb, incise, and drain an auricular hematoma, and 3) review methods for applying dressings to auricular hematomas.

**Educational Methods:**

Residents were given a five-minute in-person demonstration of how an auricular hematoma is drained using one of the models. They were then able to walk through the steps of incising, draining, and dressing the ear themselves using material found in the emergency department. The EMRAP instructional video was reviewed by the attending conducting the demonstration prior to teaching.^7^

**Research Methods:**

Eleven participants were instructed to incise and drain an auricular hematoma. The Michigan Standard Simulation Experience Scale (MiSSES) was used for anonymous feedback on the task trainer immediately after the simulation. Participants scored the simulator in five categories: Self-efficacy, fidelity, educational value, teaching quality, and the overall rating on a 5-point Likert scale, 1 = Strongly Disagree, 5 = Strongly Agree. The scale is a freely available, validated subjective-assessment tool created at the University of Michigan to standardize the evaluation of simulation-based education. In the scale, there are six domains for evaluation, and they demonstrated good internal consistency at initial psychometric testing.^8^ Variables were summarized using the mean and standard deviation.

**Results:**

The eleven participants were composed of 7 PGY 1 EM residents and 4 EM attendings. They all completed the simulation and filled out the MiSSES form. Of the residents, none had any previous experience with auricular hematoma drainage. Of the attendings, all had done one previously, but none more than 3. Using the MiSSES, the model received an overall positive rating of 5.0. The highest rating was on teaching quality (5.0) and educational value (5.0). For fidelity, the trainer received a 4.9/5.

**Discussion:**

Overall, the residents felt that this simulation was a good training tool for auricular hematoma, which is a lower occurrence but time-sensitive procedure. We created a reusable and cheap simulator model that scored high in a standard simulator’s assessment survey.

**Topics:**

Auricular hematoma, incision and drainage, cauliflower ear.

## USER GUIDE

**Table:** 

List of Resources:	
Abstract	1
User Guide	3
Appendix A: PowerPoint Presentation	8

**Learner Audience:**

This auricular hematoma task trainer is designed to instruct emergency medicine (EM) residents, fellows, and medical students. Otolaryngology trainees may also benefit from this simulator.

**Time Required for Implementation:**

The time for setup was approximately 20 minutes per ear, with an additional 40-minute set time for the glue prior to use. Multiple ears could be made simultaneously.

Learners were given a 20-minute session in groups of four to watch the demonstration and complete the simulation.

The ears are reusable; however, they may not be reusable during a single simulation session. Previous incisions can be closed using the Sil-Poxy, which applies in five minutes or less, but has an additional 40 minutes of cure time.

**Recommended Number of Learners per Instructor:**

Recommended learner ratio is 4:1

**Topics:**

Auricular hematoma, incision and drainage, cauliflower ear.

**Objectives:**

By the end of this session, learners will be able to:

1. Identify the landmarks used to drain auricular hematomas
2. Demonstrate ability to numb, incise, and drain an auricular hematoma
3. Review methods for applying dressings to auricular hematomas

### Linked objectives, methods and results

The instructor should point out the landmarks for incision and drainage (Objective 1). The skin of the auricula can necrose with pressure, and the residents should be familiar with the pinna, the helix, the anti-helix, and the conch.^9^

First, an auricular ring block is demonstrated, and then residents can replicate. Numb the greater auricular nerve, lesser occipital nerve, and auriculotemporal nerve by inserting the needle just inferior to the attachment of the ear lobe to the scalp and directing the needle towards the tragus while aspirating; normally 2 cc’s of lidocaine is injected, but injection is not recommended on this model. The needle is then redirected posteriorly and superiorly, and this process is repeated. The needle is then re-inserted just superior to the ear’s helix, where it attaches to the scalp, and is advanced towards the tragus first while aspirating; residents can simulate injecting 2 cc’s of lidocaine once again, and then redirect posteriorly and inferiorly to repeat the aspirating and injecting.^10^ To actually perform the incision and drainage, the hematoma should be incised along the posterior edge, following the curvature of the pinna. A second incision can be made along the anti-helix following the anatomic fold. (Objective 2) The hematoma should be irrigated and manually evacuated using firm pressure.^9^,^11^

After irrigating an auricular hematoma, it is essential to place a bolster pressure dressing to avoid re-accumulation of the blood. The next step can be done in one of two ways, either wrapping elastic and gauze bandages around the head, which can be demonstrated on a live participant, or by physically sewing in a bolster dressing on the model ear using 2-0 silk (Objective 3).^9^,^11^ Bolster dressings can be made using dental cotton rolls or rolling 4×4’s and applied to the ear along the contour of the helix, as one would a real patient. (see Appendix 1)

### Recommended pre-reading for instructor

- Dinces EA. How to drain an auricular hematoma. *Merck Manual Professional Edition*. Published May 2025. https://www.merckmanuals.com/professional/ear-nose-and-throat-disorders/how-to-do-ear-procedures/how-to-drain-an-auricular-hematoma
- Greywoode JD, Pribitkin EA, Krein H. Management of auricular hematoma and the cauliflower ear. *Facial Plast Surg*. 2010 Dec;26(6):451–5. Epub 2010 Nov 17. PMID: 21086231. doi:10.1055/s-0030-1267719
- Hohman MH, Jamal Z, Krogmann RJ, et al. Auricular hematoma. [Updated 2024 May 1]. In: *StatPearls* [Internet]. Treasure Island (FL): StatPearls Publishing; 2025 Jan-. Available from: https://www.ncbi.nlm.nih.gov/books/NBK531499/
- Mason J. Auricular hematoma incision and drainage with bolster compression dressing [video]. EM: RAP Medical Education YouTube channel. Published April 2024. https://www.youtube.com/watch?v=sviy_Xc7zxM

### Learner responsible content (LRC)

- Berg A, Byrne E. Simple steps to auricular hematoma drainage. NUEM Blog. Published July 26, 2016. https://www.nuemblog.com/blog/auricular-hematoma

### Additional educational materials

- Appendix A: Auricular Hematoma Model Demonstration

### Implementation Methods

Residents should be given a five-minute in-person demonstration using one of the models. (Appendix 1) They can be shown how to numb the ear with an auricular nerve block. They can then walk through the steps of incising, draining, and dressing the ear themselves using material found in the emergency department.

### List of items required to replicate this innovation

1. 2 pcs Soft Silicone Ear Model, Left and Right Flexible Fake Ear for Piercing Practice, Realistic Ear Mold for Jewelry Display Acupuncture Mannequin Learning (Beige) https://www.amazon.com/s?k=Realistic+Ear+Mold+for+Jewelry+Display+Acupuncture+Mannequin+Learning&crid=C5FK0GMIDRMH&sprefix=realistic+ear+mold+for+jewelry+display+acupuncture+mannequin+learning%2Caps%2C152&r ef=nb_sb_noss
2. Smooth-On SIL-poxy Rubber Silicone Adhesive - 0.5 oz Tube Silpoxy https://www.amazon.com/dp/B00IRC1YI0
3. J-Loop
4. 10 mL Luer Lock Syringe
5. 3-Way Stopcock Luer Lock
6. IV catheter
7. Spare thin piece of flesh-colored silicone or 1 mm silicone blank tattoo skin practice sheet https://www.amazon.com/s?k=1+mm+silicone+blank+tattoo+skin+practice+sheet&i=industrial&crid=R74L1QO1QEHN&sprefix=1+mm+silicone+blank+tattoo+skin+practice+sheet%2Cindustrial%2C183&ref=nb_sb_noss
8. Red simulated blood concentrate https://www.amazon.com/s?k=Red+simulated+blood+concentrate&i=beauty&crid=26VTW23HMS0XD&sprefix=red+simulated+blood+concentrate%2Cbeauty%2C215&ref=nb_sb_noss
9. A #10 or 15-blade scalpel
10. Hemostats
11. 2-inch bandage or 4×4’s rolled into a cylinder
12. 2-0 silk suture

### Approximate cost of items to create this innovation

It costs approximately $10.90 to make each auricular hematoma trainer.

### Detailed methods to construct this innovation

1. Prepare the Silicone Ear: Insert an IV catheter through the silicone ear, exiting at the triangular fossa. Retract the eedle, then trim the catheter tubing to approximately ½ inch in length.
  **Figure:**
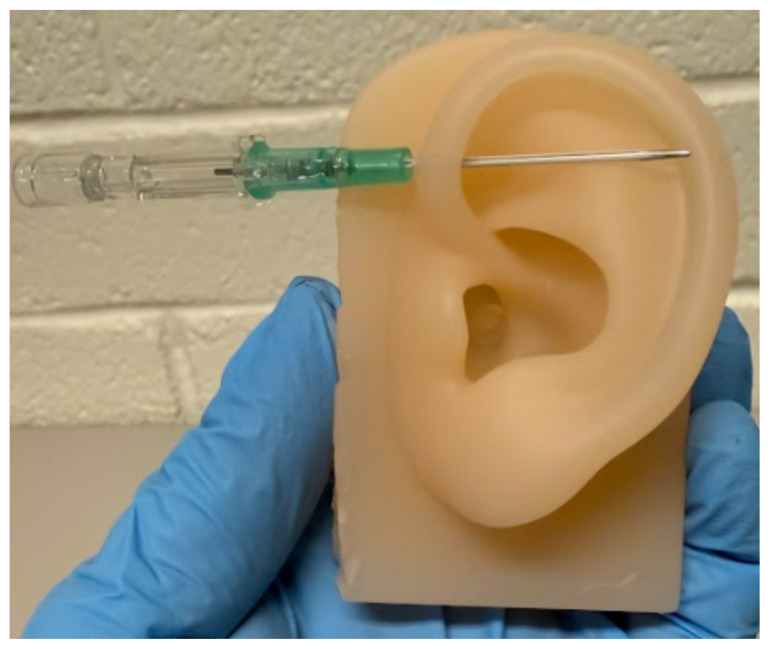
2. Secure the IV Catheter: Use Sil-Poxy adhesive to affix the catheter hub to the base of the silicone ear. Apply a small amount of Sil-Poxy at the catheter’s exit point on the ear to ensure a watertight seal. Allow to dry for 20 minutes.
  **Figure:**
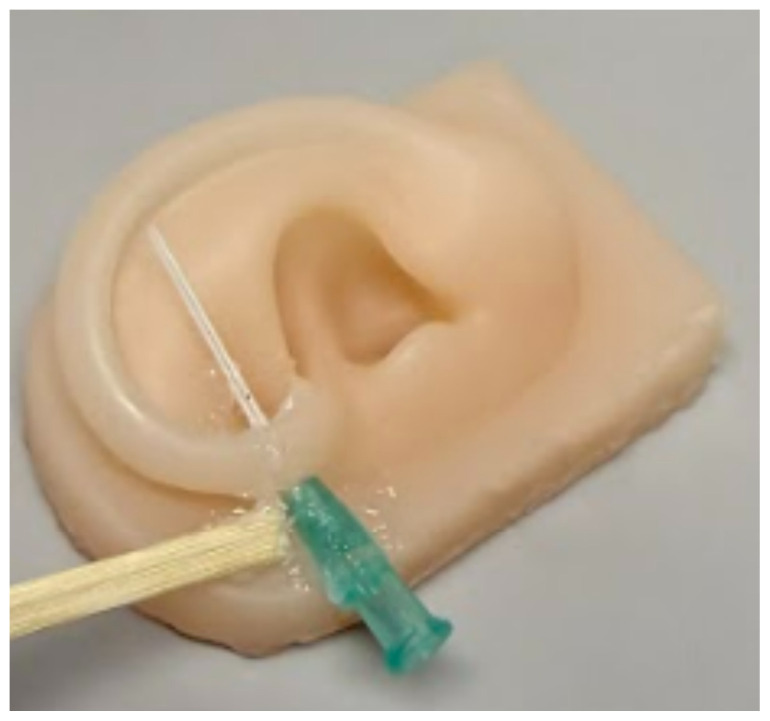
3. Create the Internal Reservoir: While the catheter is drying, cut a comma-shaped piece from a flexible silicone sheet to mimic the anatomical contour of an auricular hematoma. The piece should extend from the upper portion of the ear to just above the earlobe.
  **Figure:**
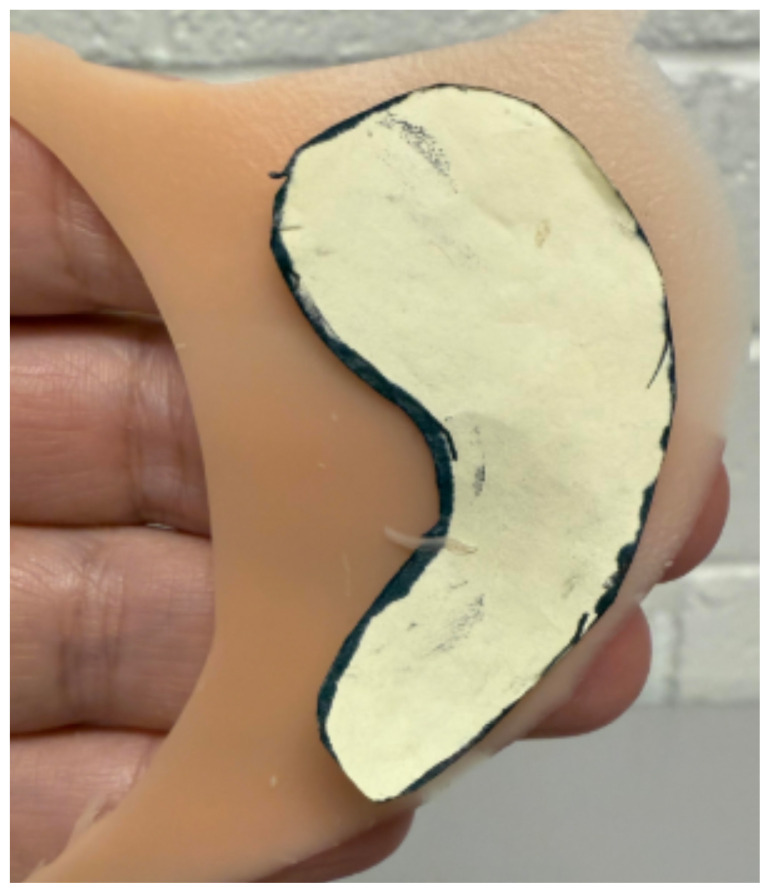
4. Adhere the Reservoir Liner: After the initial drying period, begin securing the cut shape to the ear using Sil-Poxy, ensuring the edges are fully sealed except for a gap in the center to allow for simulated blood flow. Avoid covering the end of the IV catheter during this step. Apply the adhesive in sections, allowing each segment to dry for at least 20 minutes before proceeding.
  **Figure:**
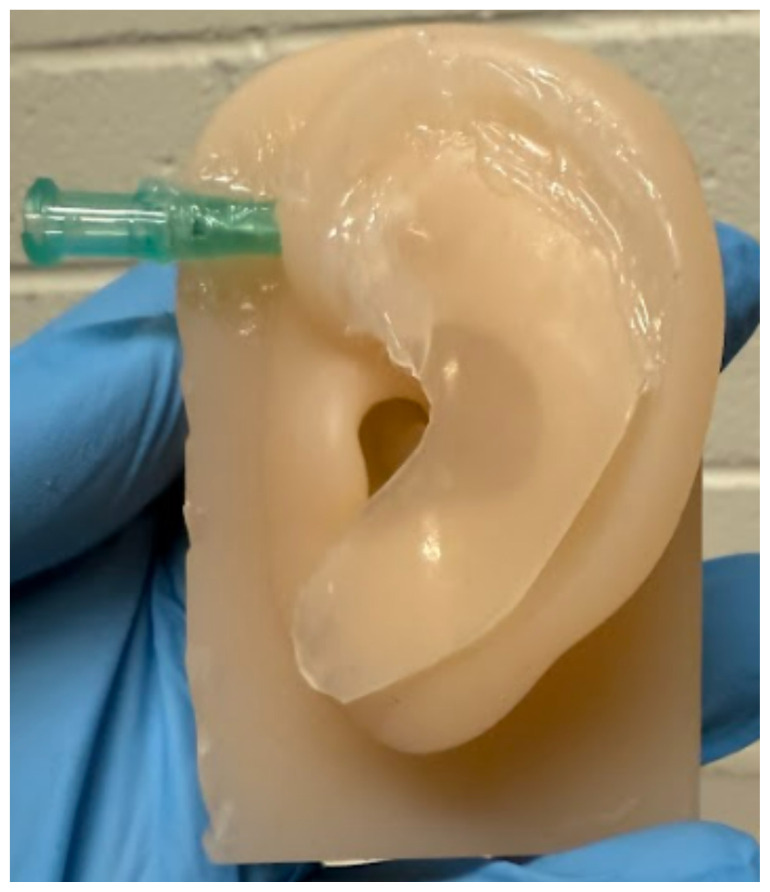
5. Prepare the Blood Delivery System: Connect a syringe to one port of a 3-way stopcock and a J-loop to another. Submerge the free end of the J-loop in a container of simulated blood and draw approximately 10 mL into the syringe.
6. Inject Simulated Blood: Attach the J-loop to the IV Catheter hub. Slowly inject simulated blood into the constructed reservoir within the ear. Monitor for leaks or incomplete seals.
  **Figure:**
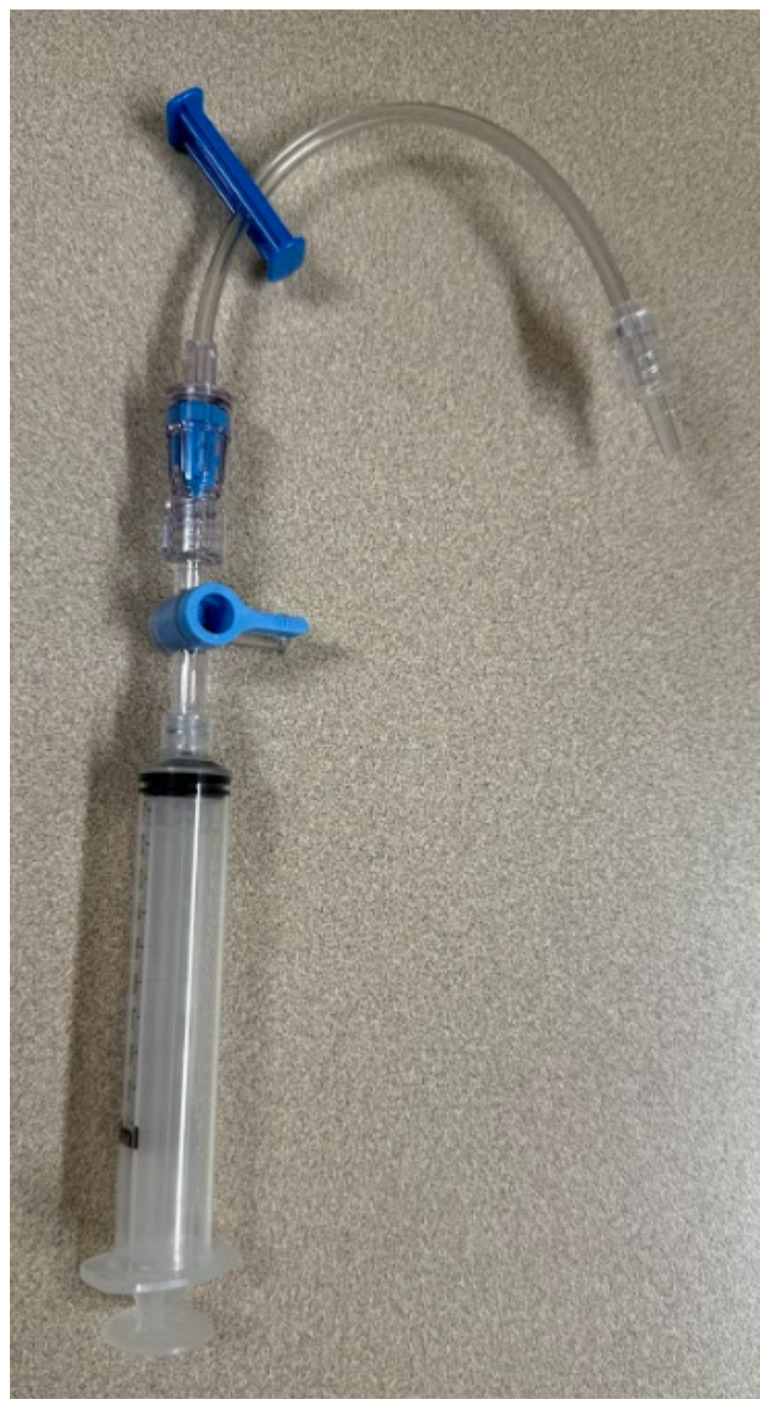

**Figure:**
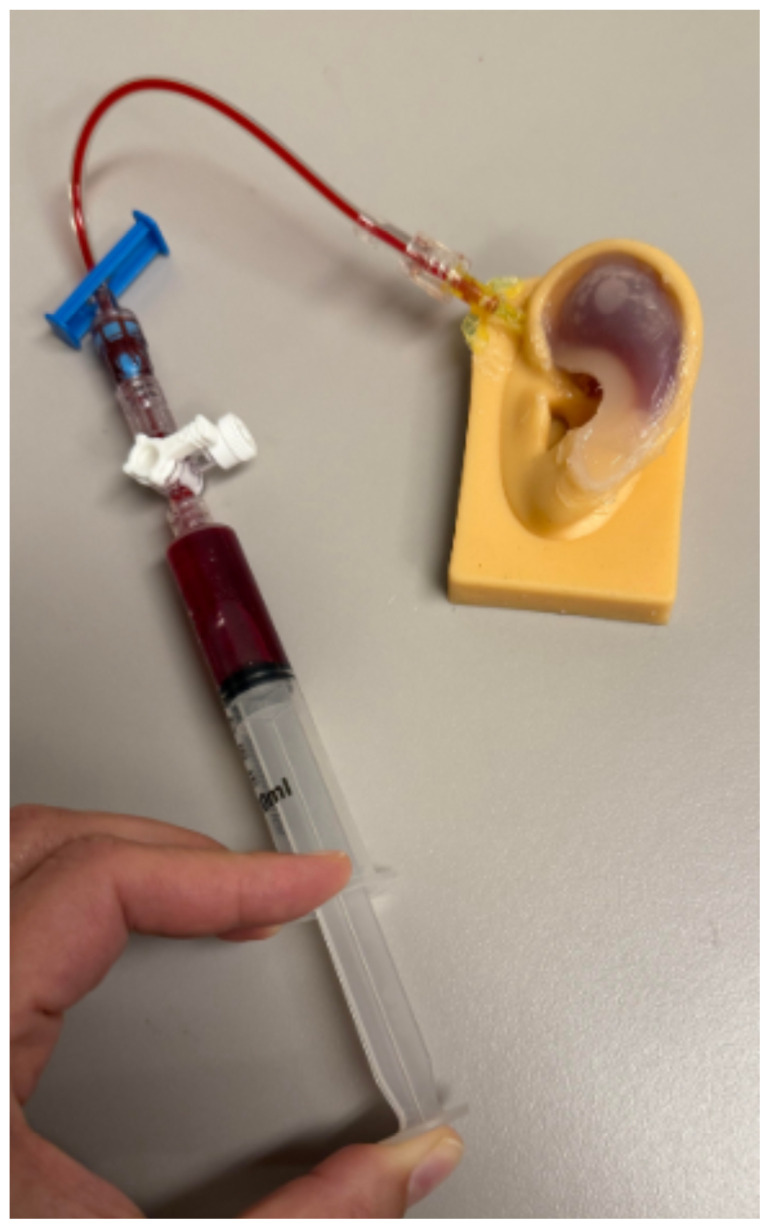
7. Seal Any Leaks: If leakage is detected, aspirate the blood back into the syringe, reinforce the area with additional Sil-Poxy, and allow it to dry thoroughly before reattempting.

### Results and tips for successful implementation

This simulation was performed during regular didactic time during an hour of “round robin” style teaching. Residents came to the table in groups of two to four and had 20 minutes to view the demonstration and perform the procedure themselves. They used a #15 blade, hemostat, and 4×4’s with various bolster dressings. Immediately after 20 minutes, residents were asked to fill out the MiSSES survey. Seven PGY 1 EM residents and four EM attendings completed the simulation and filled out the MiSSES form (Table 1). Of the residents, none had any previous experience with auricular hematoma drainage. Of the attendings, all had done one previously, but none more than three. Using the MiSSES, the model received an overall positive rating of 5.0. The highest rating was on teaching quality (5.0) and educational value (5.0) (Table 2). For fidelity, the trainer received a 4.9/5.

It was quickly recognized that the model builds up pressure on the inside with large amounts of fake blood placed, and can ‘explode’ violently when incised, so mindfulness was made to only place 1 cc or less of fake blood within the hematoma cavity at a time. Ensure that this procedure is done on chucks with paper towels present to help clean up the fake blood. Gloves would also be encouraged because the fake blood can stain hands.

The ears can also be reused by sealing the previous incisions with Silpoxy adhesive and allowing six hours to dry.

### Associated content

- Appendix A: PowerPoint Presentation

## Supplementary Information



## Figures and Tables

**Table 1:** Baseline Characteristics

Participants	Male	Female
PGY 1 Residents	1	6
Attendings	2	2

**Table 2:** 

Statement	Mean (SD) Range 0–5 0 = Don’t know 5 = Strongly Agree
**Self-efficacy**	
This simulator helped improve my knowledge	4.9 (0.3)
This simulator helped improve my confidence in performing an auricular hematoma incision and drainage	5 (0)
This simulator helped improve my skills in nerve block for ears and performing incision and drainage	4.9 (0.3)
This simulator helped improve my ability to incise and drain auricular hematomas independently	5.0 (0)
**Fidelity**	
The simulator used has adequately realistic characteristics/features	4.9 (0.3)
**Teaching Quality**	
Instructors were knowledgeable about the topic	5.0 (0)
Instructors were able to convey material in a way that was understandable to me	5.0 (0)
**Educational Value**	
The simulator is a good training tool for skills in auricular hematoma drainage	5.0 (0)
Overall rating for this simulator:	5.0 (0)

**Figure:**
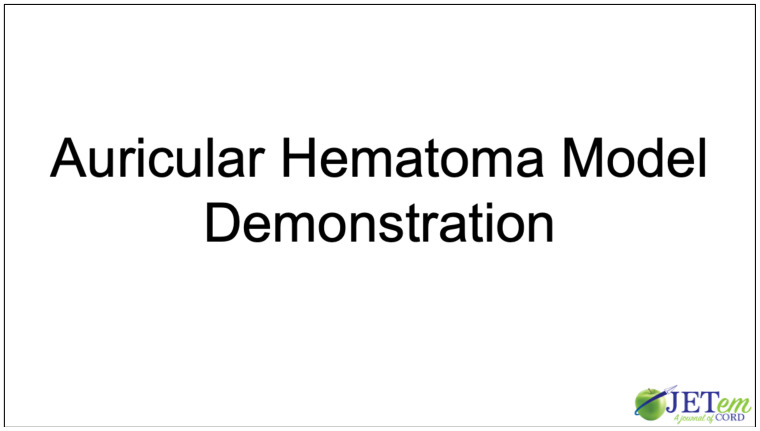
Please see associated PowerPoint file
